# Biofilm Survival Strategies in Chronic Wounds

**DOI:** 10.3390/microorganisms10040775

**Published:** 2022-04-05

**Authors:** Ida Clement Thaarup, Anne Kristine Servais Iversen, Mads Lichtenberg, Thomas Bjarnsholt, Tim Holm Jakobsen

**Affiliations:** 1 Costerton Biofilm Center, Department of Immunology and Microbiology, University of Copenhagen, 2200 Copenhagen, Denmark; icthaarup@sund.ku.dk (I.C.T.); anne.kristine.iversen@sund.ku.dk (A.K.S.I.); mlichtenberg@sund.ku.dk (M.L.); tbjarnsholt@sund.ku.dk (T.B.); 2 Department of Clinical Microbiology, Copenhagen University Hospital, 2100 Copenhagen, Denmark

**Keywords:** bacterial biofilm, transcriptomics, extracellular polymeric substances, microenvironment, animal models

## Abstract

Bacterial biofilms residing in chronic wounds are thought to have numerous survival strategies, making them extremely difficult to eradicate and resulting in long-term infections. However, much of our knowledge regarding biofilm persistence stems from in vitro models and experiments performed in vivo in animal models. While the knowledge obtained from such experiments is highly valuable, its direct translation to the human clinical setting should be undertaken with caution. In this review, we highlight knowledge obtained from human clinical samples in different aspects of biofilm survival strategies. These strategies have been divided into segments of the following attributes: altered transcriptomic profiles, spatial distribution, the production of extracellular polymeric substances, an altered microenvironment, inter-and intra-species interactions, and heterogeneity in the bacterial population. While all these attributes are speculated to contribute to the enhanced persistence of biofilms in chronic wounds, only some of them have been demonstrated to exist in human wounds. Some of the attributes have been observed in other clinical diseases while others have only been observed in vitro. Here, we have strived to clarify the limitations of the current knowledge in regard to this specific topic, without ignoring important in vitro and in vivo observations.

## 1. Introduction

The prevalence of chronic wounds is rising [1,2] and in developed countries the estimated lifetime risk is 1–2% predominately affecting the elderly population [2,3]. The terms “chronic” and “nonhealing” wounds are used interchangeably with no universally accepted definition for chronicity [4]. Depending on the literature and the type of wound, the time span for chronicity is defined between 2 weeks to 3 months [4,5,6]. The most common types of chronic wounds are vascular ulcers (venous or arterial), diabetic ulcers, and pressure ulcers present on the lower limbs [4]. The ulcers are associated with immobility, stress, reduced quality of life, amputation, severe infection, death as well as high economic costs [5,7].

There is an increasing body of evidence that suggests that bacterial biofilm aggregates play a role in the delayed healing of chronic wounds [8,9,10]. By its simplest definition, a biofilm is a group of bacterial cells imbedded in a matrix, that has increased tolerance to antimicrobials and the host defense system. It is estimated that biofilms are present in approximately ~80% [10,11] of chronic wounds as compared to only 6% of acute wounds [9]. These numbers may be underestimated as biofilms are not uniformly distributed [12] and the chance of identification will be affected by the method used as well as the sampling approach [13,14]. The bacteria found in wounds are thought to originate from the patients’ skin or other body parts such as the oral cavity or the gut, or from the outside environment [15]. In most wounds, the endogenous bacteria, i.e., the ones derived from the patients themselves, are thought to predominate [16]. Normal wound healing progresses through the phases of hemostasis, inflammation, proliferation, and remodeling in a matter of weeks to months depending on wound size [17]. However, the presence of biofilms is hypothesized to cause an exaggerated innate immune response, leaving the wound in a chronic inflammatory state with collateral tissue damage [2,18]. Proper management of biofilms in chronic wound infections is therefore believed to be key in successful healing [19].

The complete eradication of the residing biofilm is difficult to achieve due to the numerous and complex survival strategies utilized [20]. The survival strategies are believed to result from a combination of the inherent biofilm properties together with interactions between the microbes and the host environment [21]. Several reviews focus on the topic of biofilm resistance and tolerance [22,23,24]. In particular, studies that compare antimicrobial resistance in planktonic and biofilm-grown cultures in vitro are plentiful (see [25] for a meta-analysis).

## 2. Current Biofilm Research

Currently, most of the research within biofilm resistance is based on in vitro experiments or animal models which are significantly different from studying chronic wounds in patients [21,26]. Essentially, during in vitro experiments an organism (or a component thereof) is isolated from its natural environment to study its mechanisms or behaviors in detail under controlled settings. In vitro observations certainly have been invaluable in discovering cellular processes, mechanistic actions, or basic interactions between entities. It is, however, unfair to assume that findings from a test tube can be directly extrapolated to in vivo conditions and caution should be taken when attempting to translate such results. Under certain conditions in vitro culturing has been shown to better resemble in vivo bacterial transcriptomes than infected animal models [27]. Over the years, novel and sophisticated chronic wound models have been developed which aim at simulating certain features of in vivo conditions and a thorough breakdown of these models was recently reviewed [28].

While animal models do include all the components observed in the human environment, such as a complex tissue structure and a functioning immune system, several papers have concluded that data obtained from rodent models in particular, do not correlate well with what is found in humans [27,29]. Common for all animal models is that none of them recapitulate all features of human skin, healing processes, and the immune response.

Firstly, the anatomy of human skin is best resembled by porcine models. In pigs, the dermal to epidermal thickness ratio is similar to human skin [30], there is a lack of panniculus carnosus, sparse body hair (but still presence of hair follicles) and the dermis has a similar architecture although eccrine glands are not present in pigs [29]. In comparison, rodent models have thin epidermal and dermal layers, a dense hair coat and have panniculus carnosus resulting in loose skin. A very relevant consideration for chronic wound research is the mode of healing. In humans and pigs, wound closure is achieved mainly by re-epithelization, resulting in scar tissue, whereas animals that have panniculus carnosus use contraction of skin for closing wounds.

Secondly, the immune response toward infecting bacteria appears to be different between animal models. This has been thoroughly reviewed elsewhere but among other factors, the leucocyte to neutrophil ratio is widely different between mice and humans as well as the induction of both the innate and adaptive immune system [31]. In contrast, the immune system of pigs has several similarities to that of humans, with only a few disparities [32,33].

Owing to the obvious constraints of having animal models that possess the same co-morbidity development over time as seen in humans (diabetes, atherosclerosis, lifestyle diseases, etc.), no animal models are able to capture the complexity of a human wound spanning months to years without proper healing [34]. However, many models have been developed to simulate certain features of the co-morbidities such as ischemic wounds, ischemic reperfusion wounds, pressure ulcers, and diabetic wounds [35].

Lastly, there is the question of the chronicity of the induced wound in the animal or the in vitro model. The term ‘chronic’ does not have a universal definition, but usually a wound that has not healed within 12 weeks is categorized as being chronic. For ethical and practical reasons, inductions of long-lasting wounds or laboratory models, respectively, are not trivial. However, if a shorter period is used important features may remain undiscovered and the experiment may not reflect the situation in an in vivo chronic wound.

**Figure 1:**
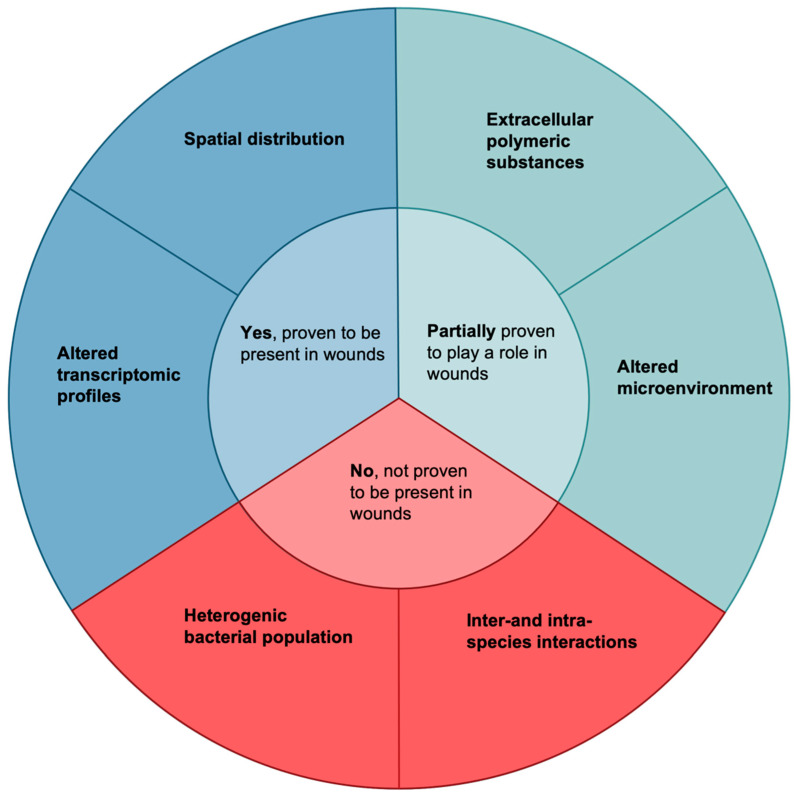
Factors that may cause increased survival of bacteria in wounds. These six qualities are all biofilm attributes or biofilm-related attributes which have been speculated to increase their survival in human wounds. Only two have been demonstrated to exist and lead to increased biofilm survival in wounds by use of clinical wound samples, namely altered transcriptomic profiles [36,37,38,39] and spatial distribution [40,41]. Two factors have been partially proven to play a role: extracellular polymeric substances [42,43,44,45,46,47,48] and an altered microenvironment [49,50,51,52,53,54,55,56,57,58,59,60,61]. Both of these factors have been shown to exist in wounds, but their role in leading to increased survival has not been proven in clinical samples. Finally, the last two factors, a heterogenic bacterial population and inter-and- intra-species interactions, have only been shown to exist in vitro or in other conditions.

Even though in vitro and animal models do not recapitulate every aspect of human chronic wounds they are still invaluable in increasing our knowledge about the mechanistic and molecular causes of non-healing wounds. In this review, we will make clear separations between data obtained from human wound samples and those observed only in vitro or in animal models to elucidate the biofilm survival strategies proven to exist and be of significance in clinical wounds. Additionally, while certain biofilm attributes may have been observed in human wound samples, their role in increased bacterial survival may not yet have been elucidated. Factors potentially involved in the increased survival of bacteria in chronic wounds are presented in Figure 1.

## 3. Altered Transcriptomic Profiles

Transcriptomic analyses are increasingly used to survey the specific gene regulations of infectious microbes. By using RNA-sequencing approaches to study gene expression in human samples, changes in, e.g., bacterial virulence, metabolism, antibiotic resistance, interactions, etc., can be studied in vivo and compared to reference strains grown in vitro. For example, a comparison of the transcriptome of *S. aureus* isolated from prosthetic joint infections and laboratory-grown cultures revealed that *S. aureus* from clinical samples expressed a change in several metabolic pathways and an increase in 131 genes encoding virulence factors, such as α-hemolysin and γ-hemolysin [36]. Similarly, a unique transcriptomic profile was identified for *P. aeruginosa* in chronic infections, where antibiotic resistance-associated genes, including efflux pumps, were upregulated in chronic wounds compared to in vitro grown cultures [37]. A metatranscriptomic analysis of diabetic foot infections identified an upregulated expression of pathways involved in the synthesis and regulation of siderophores (iron-chelating molecules) and cell-surface components (fimbria and flagellum) [38]. Recently, the first paper was published that considered both bacterial and human transcriptome in patients with diabetic foot ulcers [39]. The upregulation of genes encoding resistance and virulence together with altered metabolic pathways are thought to increase the survival potentials of microbial biofilms in wounds [37].

The challenge of collecting sufficient useful material from clinical samples for sequencing can potentially be overcome by using single-cell RNA-seq (scRNA-seq) [62,63]. The use of this technique is increasing in transcriptome studies of eukaryotic cells. However, the scRNA-seq of bacteria is in its infancy, and to date, only a few studies have been published [64,65].

## 4. Spatial Distribution

**Figure 2:**
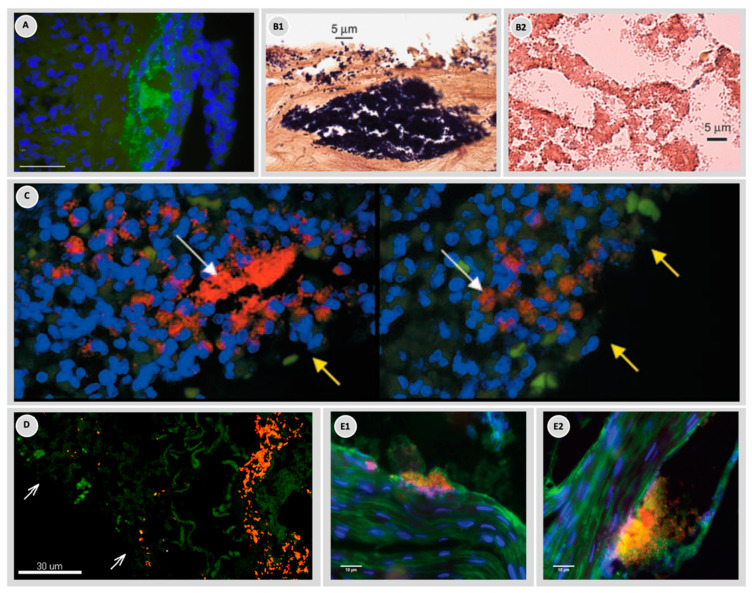
Visualization of bacterial biofilms in chronic wounds by different staining techniques and microscopy. (**A**) A chronic venous leg ulcer with *P. aeruginosa* aggregates (green) visualized by PNA fluorescence in situ hybridization (FISH) and inflammatory cells visualized by DAPI (blue) [13]. (**B1**,**B2**) Aggregates in ulcers visualized by Gram staining (**B1**) Gram-positive cocci near the surface of a pressure ulcer and (**B2**) Gram-negative rods in a diabetic ulcer [9]. (**C**) Visualization by PNA FISH of *P. aeruginosa* aggregates (red) in a chronic wound and visualization of inflammatory cells by DAPI (blue). The white arrow indicates localization of bacterial aggregates and the yellow arrow the wound surface [42]. (**D**) Aggregates of *P. aeruginosa* (red) and *S. aureus* (green) in a chronic wound visualized by PNA FISH. White arrows indicate the wound surface [40]. (**E1**,**E2**) Aggregates in chronic diabetic foot wounds visualized by a combination of FISH and Concanavalin A-conjugated Alexa Fluor 488 that binds to the biofilm matrix [43].

To better understand biofilms it is not only important to know which bacterial species are present but also the organization and distribution of bacteria within the wound. A study investigating samples from three patients with chronic wounds showed that when dividing and comparing samples from different areas of the same wound, the abundance of bacteria varied significantly [13]. Another study by Davies et al. investigated which bacteria could be identified in wounds by either culturing a surface swab or using the molecular method of a punch biopsy [66]. The results showed that more than 40% of the bacteria identified on the punch biopsy using molecular methods were not isolated by culturing the swab [66]. While the discrepancies between different diagnostic methods have been discussed before [13,67,68] and will be highlighted later, the results indicate an uneven distribution of bacteria within chronic wounds (Figure 2).

To our knowledge, only a single study has investigated the depth distribution of bacterial biofilms in chronic wounds [40]. By using peptide nucleic acid-based fluorescence in situ hybridization (PNA-FISH) combined with confocal laser scanning microscopy (CLSM), the depth distribution of *S. aureus* and *P. aeruginosa* was quantified in samples from nine chronic venous leg ulcers. The authors found a non-random distribution where *S. aureus* was primarily located 20–30 µm from the wound surface, whereas *P. aeruginosa* was primarily located 50–60 µm from the wound surface both as single species biofilms (Figure 2D). These findings were suggested as a possible explanation for previous findings of discrepancies between surface swabs and tissue samples [40]. It was speculated that the location of the biofilm aggregates could affect survival, as topically added compounds might not reach the biofilms located deep within the wound bed. This has previously been observed in a clinical study in which patients were treated with silver-containing dressings [41]. Surface swaps revealed a decrease in bacterial numbers, but the enumeration of bacteria from biopsies did not show a reduction in microbe numbers, indicating that pathogens located at a greater depth had enhanced survival. Moreover, any added compound could also be diluted to sub-inhibitory concentrations as it moves through the wound debris before it reaches its target, providing another survival benefit for microbes located deeper in the wound.

## 5. Extracellular Polymeric Substances

Extracellular polymeric substances (EPS) are present in wounds as has been demonstrated in a few studies using different visualization and staining techniques. The encasement of bacteria in EPS has been demonstrated using CLSM combined with probes targeting different exopolysaccharides [42,43,44] (Figure 2(E1,E2)). An alternative to confocal microscopy is to use scanning electron microscopy. Using this technique, micro-colonies encased in an EPS of different thicknesses were observed in samples obtained from diabetic foot ulcers [45]. From in vitro studies, we know that the EPS can modulate the microenvironment of the inhabiting microbes, while also affecting the pathogenesis of the biofilm [46,47,48]. Moreover, some studies have found that extracellular matrix components may affect the response of the polymorphonuclear leukocytes (PMNs) of the immune system [48]. In vitro analyses of EPS components have found it to consist of microbe-produced compounds such as lipids, proteins, exopolysaccharides, and extracellular DNA (eDNA), but it has been speculated that biofilms found in wounds also incorporate host material such as collagen and fibrin [46,69]. To our knowledge, a detailed investigation of EPS components from wound biofilms has not yet been carried out and it is not clear to what degree host material contributes to the biofilm matrix. It is also not clear whether host material alters the physiology and pathogenicity of bacteria in wounds. In vitro experiments have shown that different EPS components may bind or otherwise hinder the penetration of antibiotics [47,70,71,72]. For example, DNA added to an in vitro biofilm of *P. aeruginosa* can be incorporated into the biofilm matrix and increase the tolerance of the biofilm to aminoglycosides [47]. Another study found that the addition of exogenous DNA to a biofilm of *S. epidermidis* increased its survival towards vancomycin [71], but such effects have yet to be shown in human samples. A study using a murine infection model and ex vivo lung tissue from CF patients showed host eDNA to be surrounding biofilm structures rather than being inside them [73]. While the presence of EPS in chronic wounds is well established, its role in terms of bacterial persistence has still not been confirmed. However, in chronically infected cystic fibrosis lungs, the presence of the EPS component alginate has been found to have a major impact on the disease condition [74]. The presence of alginate is correlated with a poor clinical condition, increased bacterial virulence, and increased bacterial resistance [75,76]. We believe that EPS in wounds could promote similar trends as observed in cystic fibrosis, leading to increased bacterial survival.

## 6. Altered Microenvironment

The microenvironment of chronic wounds displays several changes compared to healthy skin as well as acute wounds. Specifically, pH and oxygen levels are hypothesized to affect wound healing and biofilm survival. For example, chronic wounds have been shown to display alkaline conditions (pH 7.3–8.9), whereas intact human skin has a lower pH ranging from 4–6 which is believed to serve as an important defense mechanism [49]. The pH of acute human wounds, although less studied, is found to have a short alkaline peak before progressing toward acidic conditions during healing [77,78]. Another in vivo study measured the pH of acute and chronic wounds and found that the lowering of pH was correlated with an improvement of the wound [79] and pH measurements have been proposed as a tool for predicting short-term healing [80]. The specific methods used for measuring pH could have an impact on the comparability between studies and, for instance, in a study conducted by Leveen et al. both glass electrodes and narrow range pH papers were used with no available comparisons between the methods [78].

Although the prolonged alkaline conditions in chronic wounds are not fully understood [81], they are hypothesized to be influenced by (i) the exposure of underlying tissue to a more neutral or alkaline pH [81], (ii) a continuous loss of carbon dioxide from the wound surface [78], (iii) tissue necrosis and bacterial activity [50,82], and (iv) debridement where the removal of necrotic tissue has been shown to immediately increase pH [49].

Aggravatingly, an in vitro study found an increased biofilm formation of both *P. aeruginosa* and *K. pneumoniae* in alkaline compared to acidic conditions [51]. Furthermore, an alkaline pH is hypothesized to confer a certain degree of protection to microbes, as some antiseptics have been found to have reduced activity at higher pH levels in vitro [50].

Even though the data presented above point towards a relationship between alkaline microenvironments and poor healing rates plus increased biofilm survival, the relationship is perhaps not as simple as this. A few older in vivo studies have investigated the success rate of skin grafts for chronic wounds, a procedure often used in the treatment, and points towards the opposite relationship, i.e., that skin grafting is more successful at higher pH levels [49]. These findings indicate that there is still a lot we can learn by further investigating the microenvironment of chronic wounds.

The role of oxygen in wound healing has long been recognized [52,53]. Patients with chronic wounds often suffer from circulatory impairment or diabetes along with other co-morbidities leading to low tissue oxygen [2]. Further, the overall oxygen consumption of residing microbes together with activated immune cells that consume O_2_ for their oxidative burst may locally deplete oxygen in wounds [54,55,56]. An alkaline microenvironment may further impair oxygen levels through decreased oxygen release as the dissociation of O_2_ from hemoglobin is pH-dependent [83] and a drop in pH by, e.g., 0.6 units will release 50% more oxygen at an O_2_ pressure of 20 mm Hg [78].

Oxygen levels can affect the efficacy of several antibiotics [57], and recently it was demonstrated that antibiotic lethality is directly dependent on the metabolic state of bacteria [58]. Both tobramycin and ciprofloxacin have been shown to display a significant reduction in activity in vitro in metabolically inactive biofilms due to oxygen limitations [57]. In this context, it has been shown multiple times that increasing oxygen levels lead to increased bacterial respiration, resensitization toward antibiotics [84,85], and increased oxygen levels through hyperbaric oxygen treatment are associated with an improved chance of healing [86]. Furthermore, the immune system needs oxygen to function properly and an efficient immune response towards a biofilm infection cannot be carried out without adequate oxygen availability [52]. Hence, the anoxic environment of chronic wounds may lead to increased microbial survival against antibiotics and the immune response.

Some bacterial pathogens are known to secrete toxins that target the host’s innate immune response and in particular neutralize the function of macrophages and neutrophils. This strategy by the invading pathogens impairs the early host immune response, thereby providing an opportunity for the bacteria to survive and establish an infection. Both *P. aeruginosa* and *S. aureus* produce toxins that have been demonstrated to target neutrophils. The biosurfactant rhamnolipid produced by *P. aeruginosa* under the control of the quorum-sensing system can cause the lysis of PMNs [59]. *S. aureus* has been shown to secrete a large repertoire of molecules that interfere with neutrophil recruitment and killing. Phenol-soluble modulins (PSMs) have multiple roles in the pathogenesis of *S. aureus*. They can trigger inflammatory responses and in particular PSMa peptides can trigger the lysis of neutrophils [60]. The hemolysin-a α-toxin (Hla) produced by *S. aureus* targets white blood cells [61]. By using in vivo infection models, Hla has been shown to be important in causing pneumonia and skin infections [87]. Moreover, *S. aureus* produces different leukocidins, and leukocidin AB has been reported to kill human neutrophils [88]. Data to support the production, activity, and effect of toxins on the innate immune defense are often generated by the use of animal models or cell cultures. The actual production of various toxins by bacteria in human infections has to be investigated in more detail to understand their precise role in the infectious microenvironment.

## 7. Inter- and Intra-Species Interactions

Most chronic wounds have been found to contain more than one species of bacteria with estimates varying according to the methods used to isolate the residing microbes. Using standard culturing techniques, an average of 3.8 species [89] and 3.0 species [13] per wound has been reported, whereas by using molecular techniques a study detected an average of 20.9 genera per wound [90]. The different techniques used for the detection and identification of species have limitations. Cultivation techniques are very dependent on the conditions for incubation and the number of bacteria present in the sample investigated. By using 16 s rRNA gene sequencing a significantly higher number of species will often be identified, however, the results can be biased by the amplification of naked DNA, DNA recovery yields, PCR primer specificity, and 16 s rRNA copy number with the risk of missing species and also detect species not being clinically relevant.

There is an ongoing scientific discussion as to whether wounds contain both mono- and multi-species biofilms or if the majority of biofilms comprise only mono-species despite the wound being polymicrobial. Both mixed-species biofilms [45,91], as well as mono-species biofilms [40,92,93], have been detected in tissue samples from chronic wounds and diabetic foot osteomyelitis, by the combined use of PNA-FISH and CLSM. Images of mono-species biofilm often show that several species are present in the wound with a distance between the individual aggregates. In either case, interactions between species may provide increased survival benefits for the concerned species. The missing knowledge of the presence and the ratio of different species in chronic wounds makes it difficult to design relevant in vitro and in vivo experiments, which can simulate the condition in patients.

Because of the technical difficulties in investigating interactions between bacteria in human clinical settings our current knowledge is based on various laboratory studies. Waste products secreted by *P. aeruginosa* have been shown to provide protection in biofilms produced by *S. aureus* towards certain antibiotics [94]. Thet et al. (2019) found that a co-culture of two species significantly increased their production of virulence factors compared to when grown individually [95]. Mixed species wound infections studied in a mouse model showed impaired wound healing and increased antibiotic tolerance compared to single-species infections [96]. Pastar et al. (2013) established a co-infection of microbial species in a porcine model and an increase in virulence factors was observed which resulted in delayed wound re-epithelialization, suggesting a prolonged survival of the microbes [97]. A possible synergistic interaction between the infecting bacteria in a wound may worsen the healing process. However, there is no human in vivo data to support this statement at present.

The calling distance between individual cells and aggregates has been studied in various laboratory models and the phenazines, pyoverdine, and pyocyanin produced by *P. aeruginosa* under the control of the cell–cell communication system quorum sensing have been used as indicators. The sharing distance of pyoverdine on a soft surface has been reported to be 100 μm [98], while in an in vitro CF lung model the sizes of the aggregates were shown to be essential for the possibility to interact over longer distances [99]. Aggregates consisting of up to approximately 2000 *P. aeruginosa* cells were unable to communicate with neighboring aggregates using pyocyanin, whereas larger aggregates of more than 5000 cells could interact at a distance of 176 μm [99]. The sizes of aggregates observed with PNA-FISH of sections from chronic wounds were found to range in diameter from 5 to 200 μm with a 5 to 50 μm median for the smallest and largest aggregates [26]. In addition, the aggregates observed are often distributed with distances larger than the reported possible calling distances, which questions if inter-aggregate communication is important in wounds.

## 8. Heterogenic Bacterial Metabolism

A classic hallmark of biofilms described in several papers is the heterogeneity of a microbial population which occurs within a biofilm [100,101,102]. Biofilms in vitro are often described to exhibit increased microbial tolerance due to reduced growth rates [23]. Single species subpopulations exhibiting different phenotypes can develop as a result of gradients in the local availability of nutrients and oxygen that can lead to a heterogenic distribution of growth rates. This has been observed in vitro in several studies where the reduced growth rates have been shown to increase the tolerance and survival of the biofilm [102,103]. Bacteria can also enter a state known as viable but non-culturable (VBNC), in which they cannot be cultivated as normal. This state is characterized by lower metabolic rates and increased resistance towards physical and chemical stress [104].

To our knowledge, subpopulations of bacteria displaying differential growth rates have not been proven to exist in wounds. However, a recent study that investigated microbes isolated from sputum samples from lung infections found that while samples from both acute and chronic lung infections contained biofilm, the growth rates of the microbes from chronic samples were significantly lower than those from acute infections [105]. This could indicate that slow microbial growth rates increase survivability in human infections which leads to long-lasting illnesses. Another study isolated VBNC bacteria from biofilms in central venous catheters [106], proving that VBNC bacteria can form in human infections. Although catheters present a significantly different environment for the residing biofilm, we speculate that the ability to enter a VBNC state might also occur in chronic wounds. However, subpopulations in infections most likely do not develop within individual aggregates but rather between aggregates as a function of their surrounding environment. The divergence into distinct subpopulations might increase the population-wide fitness of the microbes within a wound environment.

## 9. Perspectives and Concluding Remarks

The ubiquity of biofilms in chronic wounds is at this point a near universally accepted fact [10]. However, it has still not been proven that the presence of biofilms causes an acute infection to turn into a chronic state. While the microenvironment of chronic wounds surely seems to promote a non-healing state, the central paradigm remains as to whether the presence of biofilms exacerbates wound healing, or if the chronic wound environment generates a favorable condition for a biofilm to settle. In any case, hard-to-eradicate biofilms are present in chronic wounds but whether it is the presence of a biofilm itself, the slow growth rate of the biofilm-residing microbes, or a combination of both that leads to chronicity is not known. Recently, it was shown that the determining factor for an acute versus a chronic lung infection was not the presence of a biofilm but rather the growth rate of the microbes within the biofilms [105]. Future studies which further highlight the differences between biofilms in acute versus chronic infections might help us in our understanding of what causes chronicity and thereby provide us with potential targets for novel treatments.

We recognize that the use of animal models for studying biofilms in chronic wounds is a necessity and particularly beneficial in answering questions of causation and correlation in terms of wound healing when biofilms are present. Such studies have already been carried out and specifically show that when biofilms are added to trauma-induced wounds (e.g., punch wounds), the infected wounds heal slower than their non-infected counterparts [107,108]. However, while such studies are highly valuable, they infer little about the specific situation of a chronic wound that has been developing for weeks or months. This distinction between acute and chronic wounds has a major impact on several factors, and while it would cause great ethical concerns to induce long-lasting wounds in animal models, the limitations of studying wounds that have only been developing for a few days must be recognized.

Although this review has emphasized the need to perform research in human chronic wound infections, complete agreement in the obtained results amongst such studies is not a guarantee. An example of this can be seen in the studies of Cornforth et al. (2018) and Morgan et al. (2019), in which both studies performed genomic analysis of *P. aeruginosa* obtained from chronic wound samples [37,109]. The study by Morgan et al. found that several virulence functions were inactivated in the *P. aeruginosa* isolates, as were many biofilm formation genes [109]. In contrast, Cornforth et al. identified an upregulation of virulence factors in isolates from human wounds [37]. Perhaps this discrepancy in the results is due to the different genomic approaches used in the two studies, albeit the disparity is still striking.

In this review, we set out to illustrate the current knowledge regarding biofilm survival strategies in chronic wounds. We have presented results obtained from real-life clinical samples as well as results obtained from animal models or in vitro experiments. In doing so, we have elucidated some of the discrepancies which exist when utilizing sub-optimal methods. The recent development of technologies has made it possible to study the microorganisms in more detail to characterize for instance their strategies for survival. With different omics approaches such as transcriptomics and proteomics, a snapshot of the underlying biology in tissue or cells can be obtained at a very high resolution. These techniques in combination with the use of host material can lead to more accurate and adequate information of bacteria in the host. Our key message is that to resolve the questions regarding biofilm survival in chronic wounds, we must look to the wounds themselves. As pointed out in this review, more specified studies are needed to understand the role of biofilms in chronic wounds.

## Data Availability

Not applicable.
